# The pivotal role of DNA methylation in the radio‐sensitivity of tumor radiotherapy

**DOI:** 10.1002/cam4.1614

**Published:** 2018-06-27

**Authors:** Xueru Zhu, Yiting Wang, Li Tan, Xiaolong Fu

**Affiliations:** ^1^ Department of Radiation Oncology Shanghai Jiao Tong University Affiliated Chest Hospital Shanghai China; ^2^ Department of Cellular and Genetic Medicine Fudan University School of Basic Medical Sciences Shanghai China

**Keywords:** DNA methylation, DNA methyltransferase inhibitors, radio‐resistant, radio‐sensitivity

## Abstract

Radiotherapy is an important modality for treatment of carcinomas; however, radio‐resistance is still a difficult problem. Aberrant epigenetic alterations play an important role in cancer development. Among epigenetic parameters, DNA methylation has arguably attracted the most attention in the radio‐resistance process. To determine the role of DNA methylation in radiation resistance, several studies were conducted. We summarized previous studies on the role of DNA methylation in radiotherapy. We observed this significant role of DNA methylation in genes related to DNA repair, cell proliferation, cell cycle process, and re‐oxygenation. Furtherly, we also conclude the predictive effect of DNA methylation on tumor radio‐sensitivity and the using of DNA methyltransferase inhibitors in clinical practice. DNA methylation plays a pivotal role in the radio‐sensitivity of tumor radio‐therapy. While hyper‐methylation or hypo‐methylation of genes is related to gene functions.

## 1 INTRODUCTION

Radiotherapy is a ubiquitous environment factor and 1 of the main therapeutic methods of malignant tumors, and approximately 50% of all cancer patients receive radiotherapy.^1^ Several tumors are relatively sensitive to radiotherapy, including nasopharyngeal carcinoma, oral cancer, head and neck tumors, breast cancer, and lung cancer. However, parts of patients with above tumors are resistant to irradiation or some initially sensitive patients develop into irradiation resistance. Hence, irradiation resistance is 1 of the major obstacles that leads to loco‐regional recurrence of malignant tumors during treatment.^2^, ^3^, ^4^, ^5^, ^6^

To enhance the radiation sensitization, researchers mainly focus on improving the state of hypoxia, increasing the damage of DNA and affecting the cell cycle. Now, commonly used radio‐sensitizers are 5‐fluorouracil, platinum, and gemcitabine in clinical practice. The sensitization mechanism of 5‐fluorouracil is to inhibit the thymidylate synthetase.^7^ For platinum, its mechanism includes radiation‐induced free radicals prompting the formation of toxic platinum intermediates, inhibition of DNA repair, radiation‐induced enhancement of cell platinum absorption, and cell cycle arrest.^8^, ^9^ Via redistributing tumor cells into S phase and causing the depletion of the deoxy ATP, gemcitabine can enhance the radio‐sensitivity of tumor radiotherapy.^10^ However, these drugs have not only limited sensitization effect, but also overlapped toxicity. Recently, researchers tend to focus the study of sensitizer on epigenetics.

The role of epigenetic modifications, especially DNA methylation, has been extensively explored in the mechanism of radio‐resistance of malignant tumors. During the development of mammalian, CpG islands in DNA are methylated de novo in a tissue‐specific manner, and the patterns are fixed in subsequent cell divisions by methyltransferase activity.^11^ In general, DNA methylation is established by DNA methyltransferase (DNMTs), including DNMT1 and DNMT3a/b. The DNMTs use S‐adenosylmethionine as a methyl donor to specifically methylate the fifth carbon atom of the cytosine ring. DNA hyper‐methylation of CpG islands in promoter regions plays a major role in the transcriptional silencing of certain genes, especially tumor suppressive genes.^12^

More recently, increasing evidence supports the suggestion that dysregulated epigenetic control through DNA methylation is important for radio‐resistance of cancer cells.^13^ The early interest in the effects of DNA methylation dating back to 2002, Kim et al^14^ found that aberrant methylation of multiple CpG dinucleotides of the ataxia telangiectasia mutated (ATM) gene led to radio‐resistance in a human colorectal tumor cell line. It has been believed that DNA methylation plays a pivotal role in variety of cellular events, including the alterations in apoptosis, cell cycle progression, mitotic checkpoint regulation, and DNA repair.^15^ The mechanisms of above cellular events have been considered to mediate radiosensitive effects.^16^

**Figure 1:**
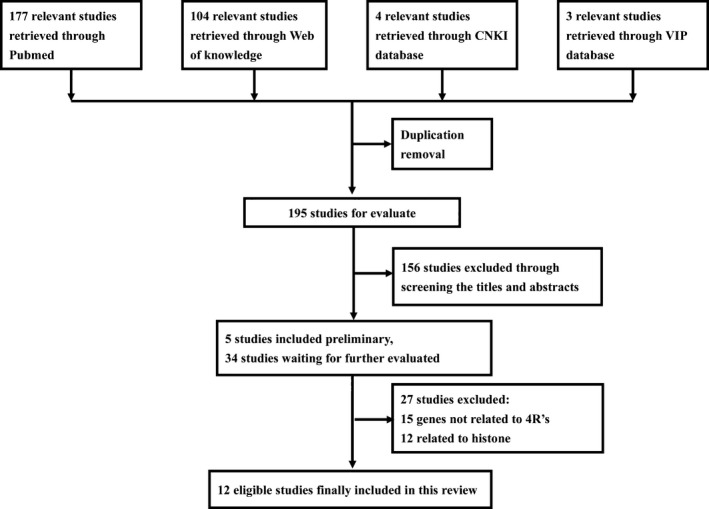
Flow chart of studies selection. 4 R's, repair, repopulation, redistribution, re‐oxygenation. CNKI, China National Knowledge Infrastructure

In this systematic review, we describe the effect of DNA methylation on radiation, the mechanisms, and applications. The flowchart of studies selection is shown in Figure 1.

## 2 RADIOBIOLOGICAL EFFECT AND THE RESPONSE RATE OF RADIOTHERAPY

Radiotherapy is a mainstay in the treatment of solid tumors, and it is proved to be effective by inducing free radical stress in tumor cells, leading to loss of reproductive integrity. Regarding clinical radiobiological effect, the optimal treatment strategy should consider damage to both tumor and normal cells.^17^ The clinical radiobiological effect is determined by 5 factors known as the 5 R's of radiobiology including DNA repair, repopulation, re‐oxygenation, redistribution, and radio‐sensitivity, which determine the curative response rate of tumor.^18^

DNA repair, repopulation, re‐oxygenation, and redistribution were known as 4 R's, which were proposed as the significant factors for the curative effect of radiotherapy of tumors. The 4 R's were discussed from the relationship of cell cycle, proliferation (G1‐S‐G2‐M), and static (G0) period. While radio‐sensitivity is the factor that reflects the response rate of tumor radiotherapy directly. Hence, we hypothesize that the DNA methylation of genes that regulate the process of 4R's plays a pivotal role in tumor radio‐sensitivity.

## 3 RADIO‐SENSITIVITY AND DNA METHYLATION OF DNA DAMAGE REPAIR‐RELATED GENES

The ataxia telangiectasia mutated (*ATM*) gene encodes a high‐molecular weight protein kinase which has been proposed to play a pivotal role in triggering appropriate cellular response to genome damage.^19^ Kim et al^14^ investigated the radio‐sensitivity of 3 hereditary Non‐Polyposis Colorectal Cancer (HNPCC) cell lines (HCT‐116, LoVo and RKO). Close inspection of the radio‐sensitivity of HNPCC cells showed that HCT‐116 cells were found to have heightened radio‐sensitivity compared to both LoVo and RKO. Further study revealed that the increased sensitivity to radiation in HCT‐116 cells was determined by the aberrant methylation of multiple GpG dinucleotides within the promoter region of *ATM* gene. Afterwards, Boston scholars Roy et al^20^ researched the role of methylation of the *ATM* promoter in the radiation sensitivity in glioma. After examining the *ATM* methylation status and radio‐sensitivity of 3 glioma cell lines, they observed the same result. For further study, they treated cells with an inhibitor of DNMT (5‐azacytidine) and showed that ATM protein in radiosensitive cells was increased and the radio‐sensitivity was decreased.

For the significant role of nucleotide excision repair (NER) in DNA repair, Chinese researchers studied the relationship between Excision Repair Cross‐complementing rodent repair deficiency 1 (ERCC1) and radio‐sensitivity of glioma.^21^ Two radiosensitive cells were with the methylated status of *ERCC1* gene, while the promoter regions of *ERCC1* gene in other 2 radio‐resistant cells were de‐methylated.

Ras Association Domain Family Member 1 (*RASSF1A*) gene encodes protein similar with the RAS effector proteins. The encoded protein was found to interact with DNA repair protein—Xeroderma pigmentosum complementation group A (XPA). This RASSF1A protein is also shown to inhibit the accumulation of cyclin D1 and thus induces cell cycle arrest.^22^

Dote et al^23^ found the enhancement of tumor cell radio‐sensitivity by the DNMT inhibitor (zebularine) in vitro and in vivo. Treating cells with zebularine, the radio‐sensitivity of 3 human tumor cells, including pancreatic carcinoma, glioblastoma, and prostate carcinoma, were more sensitive than control groups.

Due to the gene silencing effect of DNA methylation, we observed that for promoting genes of DNA repair (eg, ATM/ERCC1), DNA methylation could heighten the sensitivity of radiation to tumors, while for the DNA repair suppressive genes (eg, RASSF1A), demethylation could enhance the radio‐sensitivity of human tumor cells. Table 1.

**Table 1:** Effect of DNA methylation status of damage repair related genes on radiosensitivity

Gene	Methylation site	Tumor	Cell line	Methylation status	P‐radiosensitivity	T‐Radiosensitivity
*ERCC1*	Promoter	Glioma	MGR1	Demethylated	Resistant	‐
			MGR2	Methylated	Sensitive	‐
			SF767	Methylated	Sensitive	‐
			T98G	Demethylated	Resistant	‐
*ATM*	Promoter	Colorectal carcinoma	HCT‐116	Methylated	Sensitive	Resistant
			LoVo	Demethylated	Resistant	‐
			RKO	Demethylated	Resistant	‐
		Glioma	U87	Methylated	Sensitive	Resistant
			T98G	Demethylated	Resistant	Resistant
			U118	Demethylated	Sensitive	Sensitive
*RASSF1A*	Promoter	Pancreatic carcinoma	MiaPaca	Hypermethylated	Resistant	Sensitive
		Glioblastoma	U251	Hypermethylated	Resistant	Sensitive
		Prostate carcinoma	DU145	Hypermethylated	Resistant	Sensitive
		NPC	CNE2	Hypermethylated	Resistant	Sensitive
			SUNE2	Hypermethylated	Resistant	Sensitive

ATM, Ataxia Telangiectasia mutated; ERCC1, Excision Repair Cross‐Complementing rodent repair deficiency 1; NPC, Nasopharyngeal carcinoma; P‐radiosensitivity, primordial radiosensitivity; RASSF1A, Ras Association Domain Family Member 1A; T‐radiosensitivity, radiosensitivity after treatment with inhibitor of methyltransferase; ‐, undetected.

## 4 RADIO‐SENSITIVITY AND DNA METHYLATION OF CELL PROLIFERATION‐RELATED GENES

Serine peptidase inhibitor 5 (*SERPINB5*) gene was originally reported to act as a tumor suppressor gene in epithelial cells, suppressing the ability of cancer cells to invade and metastasize to other tissue.^24^ Maspin (mammary serine protease inhibitor) is a protein encoded by *SERPINB5* gene.^25^ Several studies have investigated the role of maspin and found its function in cell proliferation.^26^ Kim et al^27^ analyzed the global CpG methylation difference between 2 radio‐sensitivity opponent nonsmall cell lung cancer (NSCLC) cell lines. In radio‐resistant NSCLC cell line, CpG islands of *SERPINB5* gene were hyper‐methylated which was much higher than that in radiosensitive cells. Reverse transcriptase‐PCR showed higher expression of *SERPINB5* gene in radiosensitive cells compared with radio‐resistant cells. Down‐regulation of *SERPINB5* gene by small interfering RNA but not methylation inhibitor in radiosensitive cells increased radiation resistance of these cells. Meanwhile, in radio‐resistant cells, they found the hypo‐methylated status of basonuclin‐1 (*BNC1*) gene encoding finger protein basonuclin‐1 which was considered to regulate the proliferation of keratinocyte.

To investigate the role of DNA methylation inhibitor in tumor radio‐sensitivity, researchers studied 3 human tumor cell line (pancreatic carcinoma, glioblastoma and prostate carcinoma).^23^ Hyper‐methylated in cancer 1 (*HIC1*) gene is a growth regulatory and tumor repressor gene. It was detected with methylated status in above 3 radio‐resistant cell lines. And after treating with zebularine, the radiation sensitivity of these 3 radio‐resistant cell lines was enhancement.

Protein encoded by transmembrane 4 L six family member 4 (*TM4SF4*) gene is a member of the transmembrane 4 family, also known as the tetraspanin family. Most of these members are cell‐surface protein that are characterized by the presence of 4 hydrophobic domains. The TM4SF4 protein is a cell surface glycoprotein that regulates cell proliferation.^28^ In radio‐resistant lung carcinoma cell lines, TM4SF4 was highly expressed. The detection of CpG methylation status of *TM4SF4* gene showed that these cell lines were hypo‐methylated which led to the high expression of TM4SF4.^5^

Furthermore, scholars also explored the function of microRNA and methylation status in radio‐resistant nasopharyngeal carcinoma (NPC). To identify the role of microRNA 24 (miR24) in NPC radio‐resistance and the mechanism by which miR24 is regulated, Wang et al^29^ studied 4 NPC cell lines including radio‐sensitive and radio‐resistant cells. Their studies showed that miR24 inhibited NPC cell growth, promoted cell apoptosis, and suppressed the growth of NPC xenografts. Further research found that miR24‐1, 1 of the miR24 precursors, was embedded in a CpG island. Aberrant promoter DNA methylation of miR24‐1 was involved in NPC response to radiotherapy. In radio‐sensitive NPC cells, miR24‐1 was hypo‐methylated while miR24‐1 was hyper‐methylated in radio‐resistant cells.

DNA methylation status of cell proliferation‐related genes affects the radio‐sensitivity differently by their various functions. High expression of tumor proliferation suppressing gene will inhibit the proliferation of tumor cells and then induce radio‐sensitive of radiotherapy. As shown above, *SERPINB5* and *HIC1* genes were hyper‐methylated in radio‐resistant cells, while *TM4SF4* and miR24 were hypo‐methylated in radio‐resistant cells (Table 2).

**Table 2:** Effect of DNA methylation status of cell proliferation related genes on radiosensitivity

Gene	Methylation site	Tumor	Cell line	Methylation status	P‐radiosensitivity	T‐radiosensitivity
*SERPINB5*	Promoter	NSCLC	H460	Methylated	Sensitive	‐
			H1299	Hypermethylated	Resistant	‐
*HIC1*	Promoter	Pancreatic carcinoma	MiaPaca	Hypermethylated	Resistant	Sensitive
		Glioblastoma	U251	Hypermethylated	Resistant	Sensitive
		Prostate carcinoma	DU145	Hypomethylated	Resistant	Sensitive
		Breast carcinoma	MDA‐MB‐231	Hypermethylated	Resistant	Sensitive
			MDA‐MB‐435	Hypermethylated	Resistant	Sensitive
*miR24‐1*	Promoter	NPC	CNE‐1	Hypomethylated	Sensitive	‐
			CNE‐2	Hypomethylated	Sensitive	‐
			CNE‐2R	Hypermethylated	Resistant	‐
			HONE‐1	Hypermethylated	Resistant	‐
*TM4SF4*	Promoter + 5′‐UTR	NSCLC	A549	Hypomethylated	Resistant	‐
			Calu‐3	Hypomethylated	Resistant	‐

HIC1, hyper‐methylated in cancer 1; miR24‐1, microRNA 24‐1; SERPINB5, serine peptidase inhibitor, clade B, member 5; NPC, nasopharyngeal carcinoma; NSCLC, nonsmall cell lung cancer; 5′‐UTR, 5′ untranslated region; P‐radiosensitivity, primordial radiosensitivity; TM4SF4, transmembrane 4 L six family member 4; T‐radiosensitivity, radiosensitivity after treatment with inhibitor of methyltransferase; ‐, undetected.

## 5 RADIO‐SENSITIVITY AND DNA METHYLATION OF CELL CYCLE‐RELATED GENES

To explore the mechanism of resistance to head and neck squamous cell cancer (HNSCC) radiotherapy, Chen et al^3^ analyzed the DNA methylation of cyclin D2 (CCND2) in 2 counterpart HNSCC cell lines. This cyclin forms a complex with cyclin‐dependent kinase 4 (CDK4) or cyclin‐dependent kinase 6 (CDK6) and acts as a regulatory subunit of the complex, whose activity is essential for cell cycle G1/S transition.^30^ The study showed that CCND2 was hyper‐methylated in radio‐resistant cell line, while in the radiosensitive cells was hypo‐methylated. After treating with 5‐aza‐2′deoxycitidine, a DNMT inhibitor, radio‐resistant cells were more sensitive to radiation.^3^

Fragile histidine triad (*FHIT*) gene regulates G2/M checkpoint and is located in a fragile chromosome site (3p13.2), which would likely be damaged by ionizing irradiation, Lin et al^6^ selected it for study. In oral carcinoma cell lines, the promoter of *FHIT* gene was hyper‐methylated in radio‐resistant cells. In radio‐sensitive cells, the methylation status of *FHIT* gene was inverse. Further in vivo study showed that 5‐aza‐2′deoxycitidine significantly re‐sensitized radio‐resistant oral cancer cell xenograft tumors. The S100 calcium binding protein A6 (*S100A6*) gene encoded protein also involves in the regulation of cell cycle progression. In NSCLC, promoter of this gene was hyper‐methylated in radio‐resistant cell line and hypo‐methylated in radio‐sensitive cell line.^27^

In NPC, researchers analyzed 2 cell cycle‐related genes including reprimo (*RPRM*) and cyclin‐dependent kinase inhibitor 2A (*CDKN2A*). *RPRM* is a gene located at human chromosome 2q23, whose expression in conjunction with p53, along with other genes induced by p53, is associated with the arrest of cell cycle at the G2 phase.^31^ *CDKN2A* is a gene located at chromosome 9, band p21.3. The gene codes for 2 proteins including the INK4 family member p16 and p14arf. Both act as tumor suppressors by regulating the cell cycle.^32^ In both radio‐resistant NPC cell lines, *RPRM* gene was hyper‐methylated and *CDKN2A* gene was hypo‐methylated. Treating with 5‐aza‐2′deoxycitidine also enhanced the radio‐sensitivity of both radio‐resistant cell lines.^2^

Radio‐sensitivity of different division cycles is not same. Cells in S phase are resistant to irradiation, while cells in M and G2 phases are sensitive to irradiation. Treating with radiotherapy, cells in sensitive phase such as phase M or G2 are selectively killed.^20^ As shown in studies, in radio‐resistant tumor cell, genes prompting cell cycles to G2/M are hyper‐methylated. Thus, these genes are silenced, and the encoded proteins are with low expression (Table 3).

**Table 3:** Effect of DNA methylation status of cell cycle related genes on radio‐sensitivity

Gene	Methylation site	Tumor	Cell line	Methylation status	P‐radiosensitivity	T‐Radiosensitivity
*CCND2*	Promoter	HNSCC	SCC‐61	Hypomethylated	Sensitive	Sensitive
			rSCC‐61	Hypermethylated	Resistant	Sensitive
*FHIT*	Promoter	Oral carcinoma	OML1‐P	Hypomethylated	Sensitive	Sensitive
			OML1‐R	Hypermethylated	Resistant	Sensitive
*S100A6*	Promoter	NSCLC	H460	Hypomethylated	Sensitive	‐
			H1299	Hypermethylated	Resistant	‐
*RPRM*	Promoter	NPC	CNE2	Hypermethylated	Resistant	Sensitive
			SUNE1	Hypermethylated	Resistant	Sensitive
*CDKN2A*	Promoter	NPC	CNE2	Hypomethylated	Resistant	Sensitive
			SUNE1	Hypomethylated	Resistant	Sensitive

CCND2, cyclin D2; FHIT, fragile histidine triad; HNSCC, head and neck squamous cell cancer; P‐radiosensitivity, primordial radiosensitivity; RPRM, reprimo; CDKN2A, cyclin dependent kinase inhibitor 24; NPC, nasopharyngeal carcinoma; NSCLC, nonsmall cell lung cancer; S100A6, S100 calcium binding protein A6; T‐radiosensitivity, radiosensitivity after treatment with inhibitor of methyltransferase; ‐, undetected.

## 6 RADIO‐SENSITIVITY AND DNA METHYLATION OF RE‐OXYGENATION‐RELATED GENES

Hypoxia Inducible Factor (HIF‐1 α) is a stress responsive transcription factor, which regulates gene expression required for hypoxic adaptation. High expression of HIF‐1 α is significantly associated with radio‐resistance.^33^ Up to date, there are few studies on the relationship between DNA methylation of oxygenation‐related gene and radio‐sensitivity. Although previous study indicated that hypoxia may be an important but not the main factor leading to the failure of tumor radiotherapy, recent study found that changing the hypoxia status could alter the radio‐sensitivity. The study utilized the prolyl‐hydroxylase inhibitor dimethyl‐oxalylglycine (DMOG) to elevate HIF‐1 α levels in mouse embryonic fibroblasts (MEFs) and demonstrated that DMOG function as radio‐protector by increasing HIF‐1 α protein levels. Further study showed that depletion of Suv39 h1 histone H3 methyltransferase reduced the ability of DMOG to protect cells from radiation damage, implicating increased histone H3 methylation in the radioprotection of cells.^34^ Thus, we thought that hyper‐methylation may affect tumor radio‐sensitivity by silencing re‐oxygenation‐related genes.

## 7 THE PREDICTIVE ROLE OF DNA METHYLATION IN TUMOR RADIO‐SENSITIVITY

In tumor cells, abnormal gene expression may result from variant in DNA copy number, sequence mutation or epigenetic changes. Tumor cells always show major disruptions in DNA methylation profiles including aberrant hyper‐methylation and hypo‐methylation of specific genes or global genome.^12^ Previous studies have shown that DNA methylation of genes may predict the radio‐sensitivity of tumor radiotherapy.

According to previous studies and gene silencing role of DNA hypermethylation of CpG islands at promoter regions, we suppose that DNA methylation affects radio‐sensitivity through gene silencing. For example, DNA damage repair prompting genes are hyper‐methylated in radio‐sensitive tumor cells. Hyper‐methylation of these genes results in the low expression of encoded proteins which are essential to DNA repair. Then damaged tumor cells could not repair effectively and timely, so tumor cells are sensitive to radiotherapy.

In addition, to examine the genome‐wide epigenetic control of radio‐resistance, researchers performed whole‐genome analysis of CpG methylation in normal and tumor lung cells.^27^ They found 1091 methylated differential genes between radio‐sensitive and radio‐resistant lung cells. Further studies indicated that the differences may be critical to epigenetic regulation of radio‐sensitivity in lung cancer cells. Thus, DNA methylation could be the predictive biomarker for tumor radio‐sensitivity.

## 8 APPLICATION OF DNA METHYLTRANSFERASE INHIBITORS IN CLINICAL PRACTICE

DNMT inhibitors are drugs that inhibit the DNMT which is function as the initiation and maintenance of DNA methylation.^35^ Above, we have referred that DNMT inhibitors treating with radiotherapy in cells and animal models can enhance the radio‐sensitivity of original radio‐resistant tumors. Yet, in clinical practice of tumor radiotherapy, the use of DNMT inhibitors is limited. The most commonly studied drugs accompany with irradiation treatment are 5‐azacytiding (5AC), zebularine, and 5‐aza‐2′‐deoxycytidine (decitabine). Among these, 5‐azacytiding and 5‐aza‐2′‐deoxycytidine have been approved by the US Food and Drug Administration (FDA) in 2004 and 2006, respectively, while zebularine is using in preclinical studies. Both drugs of 5‐azacytiding and 5‐aza‐2′‐deoxycytidine are approved for treating myelodysplastic syndromes, acute myeloid leukemia, and other myeloid syndromes.^36^ But in tumor radiotherapy, they have not been recommended. Considering the toxicity of drugs, 5‐azacytidine with poor selectivity, which can incorporate into both RNA and DNA, has strong toxicity. The rank of toxicity among these drugs is 5‐azacytiding > 5‐aza‐2′‐deoxycytidine > zebularine. Other studied compounds are in their earlier stages, such as RG108 and EGGG.^37^

## 9 CONCLUSION

Our knowledge about the role of DNA methylation in radio‐sensitivity is mainly from in vitro and in vivo experimental system. The understanding of this pivotal role in human body is limited. Therefore, these studies should be interpreted with caution. Now, there are few studies on the relationship between hypoxic‐related genes’ methylation and radio‐sensitivity. Maybe, further researches are needed. In addition, 2 FDA approved drugs are limited in clinical practice due to their toxicity. While the low toxicity drug zebularine has not been approved to use in clinic.

According to the summary of these studies, we can conclude as follows. First, the methylation level of DNA damage repair related genes is higher than that of genes negative to DNA repair. Second, except *CDKN2A* and *TN4SF4*, cell cycle and cell proliferation‐related genes were hyper‐methylated in radio‐resistant cell lines, while the genes in radio‐sensitive cells were hypo‐methylated. *CDKN2A* gene which regulates the arrest of cell cycle at the G2 phase is hypo‐methylated in radio‐resistant nasopharyngeal carcinoma cells. The TM4SF4 protein, a cell surface glycoprotein that regulates cell proliferation, is also hypo‐methylated in radio‐resistant nonsmall cell lung cancer cell lines. Thus, the methylation level of genes in tumor cells is related to their functions. From previous studies, the pivotal role of DNA methylation in tumor radio‐sensitivity is obviously clear. And radio‐resistant tumor cells and animal models with hyper‐methylation status can be reversed into radio‐sensitive ones.

Due to the prospective results of tumor cells and animal model experiments, we will validate this pivotal role of DNA methylation in human tumor specimens. Furthermore, with the development of emerging circulating tumor cell (CTC) technology,^38^ we could detect the methylation status of radio‐sensitivity‐related genes in CTC. Furtherly, comparing the methylation status of human specimen with CTC to analyze the sensitivity and specificity of CTC technology. Supposing that CTC technology could be used to detect DNA methylation in radio‐resistant tumors, and then, it is possible to make clinical practice more convenient and feasible. In addition, studies have shown that the inhibitor of DNMT enhance the radio‐sensitivity of tumor cells. These experimental results can promote the development and application of related drugs in clinical practice of tumor radiotherapy, which will bring gospel to patients.

## CONFLICT OF INTEREST

None declared.
